# Just What I See? Implications of Congruence Between Supervisors’ and Employees’ Perceptions of Pay Justice for Employees’ Work-Related Attitudes and Behaviors

**DOI:** 10.3389/fpsyg.2020.02069

**Published:** 2020-09-09

**Authors:** Sofia Malmrud, Helena Falkenberg, Constanze Eib, Johnny Hellgren, Magnus Sverke

**Affiliations:** ^1^Department of Psychology, Stockholm University, Stockholm, Sweden; ^2^Department of Psychology, Uppsala University, Uppsala, Sweden

**Keywords:** organizational justice, justice enactment, pay justice, perceptual congruence, performance-based pay, work attitudes, job performance

## Abstract

Perceiving a pay system as just has been suggested to be a precondition for individualized pay to have a motivating effect for employees. Supervisors’ enacted justice is central for understanding the effects that pay setting can have on employee attitudes and behavior. Yet, enacted justice has received little research attention, in regard to both organizational justice and pay-related topics. This study examines the effects of employees’ perceived pay justice and supervisors’ enacted justice, as well as the degree of congruence, on employees’ work-related attitudes and behaviors. Questionnaire data from employees (*N* = 566) matched with data from their pay-setting supervisors (*N* = 208), employed in a Swedish manufacturing company, were analyzed. Results of polynomial regression with response surface analysis show that employees’ perceptions of pay justice were important for their work-related attitudes and behaviors and that supervisor–employee congruence regarding pay justice was positively related to employees’ attitudes and behavior, particularly when the ratings concerned high levels of justice. The results not only highlight the importance of developing a pay system that is perceived as just by employees but also emphasize the importance of reaching a congruence between supervisors’ and employees’ perceptions of high fairness, as this has positive implications for employees’ attitudes and behaviors.

## Introduction

Organizations need to attract knowledgeable and skillful employees and to retain those who are satisfied and motivated to perform. Individualized performance-based pay setting is a tool that organizations use to try to increase the motivation and performance of its employees. Although the possible specific effects of individualized pay setting are much debated in research (e.g., Gerhart and Fang, 2014), it has been utilized to improve employee performance as well as to attract and retain skilled and desirable personnel (Heneman et al., 2000). In order for a pay system based on individualized pay to bring about the desired results, research indicates that it is essential that employees see a clear connection between their work output and the pay and pay raises they receive (Lawler, 2000), and that they perceive the pay setting to be fair (Cropanzano and Greenberg, 1997; Rynes et al., 2004; Andersson-Stråberg et al., 2007). The functioning of individualized performance-based pay setting is mainly the responsibility of the supervisors, whose actions shape the employees’ justice perceptions regarding the pay process. Social exchange theory, which distinguishes between economic and social exchanges (Blau, 1964), can be usefully applied in this regard, as individualized pay setting encompasses more than merely an economic exchange; it rests to a large extent on a social exchange between the supervisor and the employee. Fairness in regard to individualized pay setting thus concerns not only how just an employee perceives his or her pay to be but also how just the pay-setting process as a whole is, including the treatment and actions of the supervisor.

There is a considerable amount of research that has found that perceptions of organizational justice are important for the work-related attitudes and behaviors of employees (Cohen-Charash and Spector, 2001; Colquitt et al., 2001, 2013; Rupp et al., 2014). Research has also shown that the decisions and actions of supervisors shape the employees’ justice perceptions and that the supervisor is the organization’s “face” of justice (Karam et al., 2019)—at least in the context of individualized pay setting. The majority of previous research has focused on employees’ justice perceptions, while less focus has been placed on supervisors’ perceptions of how just they act—their enacted justice—as has been the case in both justice research (Scott et al., 2009; Graso et al., 2019) and compensation research (Beer and Cannon, 2004; Levy and Williams, 2004). To understand the consequences of justice, it is important to take into account both the one who is experiencing justice, the employee, and the one who is enacting justice, the supervisor (Scott et al., 2009; Graso et al., 2019). In other areas of organizational psychology (e.g., leadership, trust, learning), it has been shown that the level of congruence between supervisors’ and employees’ evaluations has considerable influence on employee outcomes (Bashshur et al., 2011; Tafvelin et al., 2017; Carter et al., 2018). It is even more seldom that studies examine both the employee and the supervisor perspectives of justice. It is implicitly built into individualized pay setting that the supervisor and the employee should have a shared view—and reach an agreement—on how well the employee has performed and, thus, what size of pay raise the performance warrants. Since research examining supervisors’ justice enactment along with the employees’ perceptions of justice has been limited, especially concerning pay setting, the significance of the roles of pay justice perceptions and of employee–supervisor congruence regarding pay justice for employees’ work-related attitudes and behavior is unclear.

**FIGURE 1:**
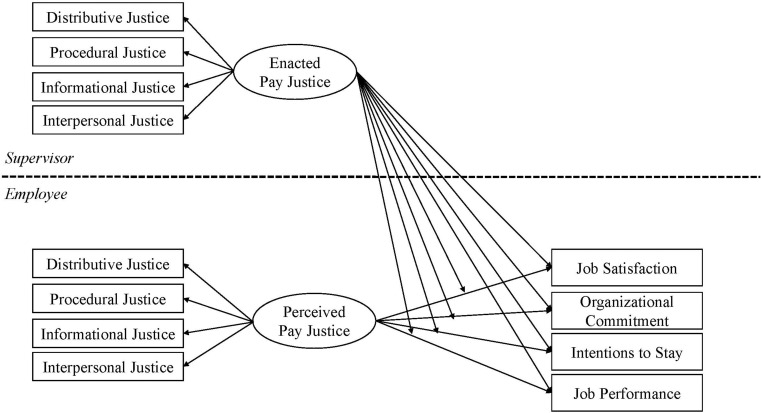
Proposed theoretical model.

The present study therefore aims to contribute to an increased understanding of the importance of pay justice and employee–supervisor congruence for individualized pay setting. More specifically, the study aims to investigate: (1) the impact of perceived pay justice on employees’ job satisfaction, organizational commitment, intention to stay, and job performance; (2) the effects of supervisors’ enacted pay justice ratings on these outcomes; and (3) whether the degree of congruence between employees’ perceived pay justice and their supervisors’ enacted pay justice can affect these outcomes. Figure 1 displays the proposed theoretical model.

### Nature of Justice

Research on organizational justice has developed over time. This research initially focused mainly on perceived justice in relation to the distribution of resources, such as pay (i.e., distributive justice; Adams, 1965; Deutsch, 1975). Justice research subsequently expanded to relate to various rules for how resources should be distributed (i.e., procedural justice; Thibaut and Walker, 1975; Leventhal, 1980), followed by a focus on how the various rules and the system encompassing the rules affect employees (i.e., interactional justice; Bies and Moag, 1986). Interactional justice has since been divided into two dimensions—informational justice (how employees are informed about how resources are to be distributed) and interpersonal justice (how the supervisors treat the employees in relation to resource distribution) (Greenberg, 1993; Colquitt, 2001).

While it is common to treat justice as consisting of four separate dimensions (Colquitt, 2001; Colquitt and Rodell, 2015), and even though it is theoretically possible to measure these four distinct dimensions of justice, there is reason to assume that the consequences of justice perceptions depend on a general conception of justice (Lind, 2001; Ambrose and Schminke, 2009). Along these lines, it has been suggested that employees form a general conception of justice by making a holistic judgment based on the available information (Greenberg, 2001). It may be difficult for employees to differentiate among the different aspects of justice such that they, rather, react to and act according to their overall conception of justice (Shapiro, 2001). It is therefore likely that employees, in practice, will conceive of justice in an overall way, in which the various aspects of justice are encompassed (Greenberg, 2001). It has also been suggested that measuring overall justice may produce more accurate and consistent results regarding how employees’ perceived justice affects their attitudes and behavior (Ambrose and Schminke, 2009; Holtz and Harold, 2009). In line with this, previous research has found support for modeling justice as an overall factor reflecting the four justice dimensions, in regard to both organizational justice (Ambrose and Schminke, 2009; Holtz and Harold, 2009) and pay justice (Choi and Chen, 2007).

### The Role of Perceived Pay Justice

Compensation research has found that it is not only how much an organization pays its employees, but also the pay system itself as well as employees’ perceptions of the pay setting, that has an impact on the work-related attitudes and behaviors of employees (Levy and Williams, 2004). If organizations want their employees to accept and support individualized pay systems, it is crucial that the outcomes of the pay system and the pay distributions are perceived as fair, as individualized pay setting has been shown to be more effective and acceptable when the process and the supervisor’s treatment are considered to be fair (Van Dijke et al., 2009; Kim, 2016).

In an individualized pay-setting system, both the way in which employees perform their work and the quality of their work typically form the basis of their performance assessment. Individualized pay setting is a continuing annual process—in which the next year’s pay process follows on the heels of the previous year’s process. Employees’ views of how well their supervisor has properly recognized and rewarded their performance during the present year’s pay-setting process, according to social exchange theory (Blau, 1964), should affect how willing they are to maintain their job performance the following year. Cropanzano and Mitchell (2005, p. 876) defined social exchange as “[a] process [that] begins when at least one participant makes a ‘move’ and if the other reciprocates.” Also according to social exchange theory, employees will be more inclined to reciprocate via their attitudes and behaviors toward the organization if they feel that they have been treated fairly (Blau, 1964). While individualized pay setting involves an economic exchange in the form of pay raises for good performance, it also involves a social exchange based on performance appreciation and recognition, in which the supervisor signals the value of the employee’s performance through rewards (Rupp and Cropanzano, 2002).

Perceptions of justice in regard to pay setting have been found to be associated with a number of employee work-related attitudes and behaviors. Perceiving that one’s pay system is fair can increase an employee’s motivation, work-related attitudes, and performance (Folger and Konovsky, 1989). Justice perceptions have almost exclusively been studied based on the various justice dimensions, and the findings indicate that employees who perceive a higher degree of justice demonstrate higher degrees of job satisfaction, organizational commitment, and job performance, as well as a stronger desire to remain working in the organization (Cohen-Charash and Spector, 2001; Colquitt et al., 2001, 2013; Rupp et al., 2014). Similar findings have also been reported in regard to justice in pay setting in particular (Sweeney, 1990; Tekleab et al., 2005; Andersson-Stråberg et al., 2007). However, overall justice has rarely been studied when it comes to pay setting outside of a Chinese context (Choi and Chen, 2007; Wu et al., 2013). For instance, in one of these studies (Wu et al., 2013), it was found that perceived pay justice predicted future performance in that low-level performers who perceived that their pay setting was fair subsequently performed at a level equal to that of the high-level performers who had perceived their pay setting to be unfair, highlighting the important role of perceived justice in pay setting. In sum, based on social exchange theory and prior research on the importance of organizational justice for employee attitudes and performance, the following hypothesis about the association between justice perceptions in pay setting was formulated:

*Hypothesis 1*: Higher levels of employee perceived justice in regard to pay setting are associated with higher levels of employee (a) job satisfaction, (b) organizational commitment, (c) intention to stay, and (d) job performance.

### The Role of Enacted Pay Justice

Previous research indicates that supervisors are an important source for employees’ perceptions of justice (Karam et al., 2019). In addition, other meta-analyses suggest that employees’ work-related attitudes and behavior appear to be more strongly affected by how they are being treated by their supervisors as compared to perceptions of justice regarding the organization as a whole (Colquitt et al., 2013; Rupp et al., 2014). Enacted justice reflects the degree to which supervisors follow or break justice rules (Graso et al., 2019). Despite the fact that supervisors may constitute a key source of justice at the workplace (Cropanzano et al., 2001), research on supervisors’ enacted justice has been limited (Graso et al., 2019), especially such research involving performance-based pay. Following the call for a greater focus on enacted justice (Scott et al., 2009), there has been an increase in studies examining actor-focused justice. However, most studies have focused on why supervisors act fairly and other characteristics of supervisors (Patient and Skarlicki, 2010; Brebels et al., 2011; Huang et al., 2017), and there is a need for further research on the potential consequences of justice enactment.

The pay-setting process occurs in various stages (e.g., continual feedback, performance assessment, pay discussion, and pay setting) that all could influence employees’ perceptions of the pay setting and, in the long run, also their work-related attitudes and behaviors. It is often supervisors who have the assignment to carry out all of these stages. While there is a large body of research showing that employees’ perceptions of supervisors’ behaviors influence work-related outcomes among employees (Karam et al., 2019), it is less clear how supervisors’ perceptions of their own behavior relate to employee outcomes. However, a social exchange requires that “something has to be given and something returned” (Cropanzano and Mitchell, 2005, p. 876), and it has been suggested that justice enactment may be one of the supervisor’s most important contributions to this transaction (Koopman et al., 2019). In turn, employees might be more encouraged to reciprocate with beneficial work attitudes and behaviors. In a study on understanding the motives of supervisors to engage in justice enactment, one of the main motives was suggested to be of instrumental nature, such as to influence the behavior of subordinates (Scott et al., 2009). The idea is that managers may want to adhere to justice rules in order to increase motivation and performance from subordinates. In other words, supervisors may provide justice enactment to their employees, and the latter return this behavior with reciprocal actions, such that a high-quality relationship is formed. Therefore, we argue that supervisors’ enacted justice is associated with work-related attitudes and behaviors of the employees. The following hypothesis was formulated:

*Hypothesis 2*: Higher levels of supervisor-enacted justice in regard to pay setting are associated with higher levels of employee (a) job satisfaction, (b) organizational commitment, (c) intention to stay, and (d) job performance.

### The Role of Perceptual Congruence

Central to social exchange theory is that the exchange between employee and supervisor concerns a relationship based on trust and loyalty that develops over time (Cropanzano and Mitchell, 2005). In order for this relationship to develop, both parties need to contribute to the exchange. Therefore, it is relevant to look not only at the individual effect of employees’ justice perceptions and supervisors’ justice enactment but also at the interplay between these two aspects. There are several concepts for describing the nature of consensus or congruence between supervisor and employee, such as perceptual congruence (Hatfield and Huseman, 1982; Turban and Jones, 1988), perceptual distance (Bashshur et al., 2011; Tafvelin et al., 2017), and self–other rating agreement (Atwater et al., 1998).

When supervisor and employee agree in their justice evaluations regarding pay setting, this could be indicative of a well-functioning social exchange relationship between the two parties. When employees have a supervisor who behaves in such a way that aligns with their perceptions, they likely detect the positive signals, and this reinforces their beliefs and perceptions. Also, the supervisor can take actions that are appropriate in the eyes of employees (Bashshur et al., 2011). When supervisors and employees disagree, however, this likely creates misunderstanding and conflict and results in a loss of resources (Tafvelin et al., 2017).

It should be noted, though, that supervisor–employee congruence need not necessarily be beneficial, since the congruence could mean that both the supervisor and the employee evaluate the level of justice to be poor rather than good. More specifically, it has been argued (Yammarino and Atwater, 1993) that positive outcomes for the individual and the organization increase when there is a congruence between supervisor and employee (especially when they agree on things being good) and lessen when supervisors rate their actions as better, and that it could go in either direction when supervisors rate themselves lower in comparison. When supervisors perceive that they have acted in a fair manner—and their employees’ justice perceptions are congruent with their appraisal—more positive outcomes may occur for the employees and for the organization; however, cases in which supervisors and their employees have congruent views on the supervisor not having performed well are more likely to be associated with less positive outcomes (Atwater et al., 1998).

Although there are few studies that have investigated justice perceptions and justice enactment as related to one another (Zapata et al., 2013; Huang et al., 2017; Koopman et al., 2019), there are no studies, that we are aware of, that have examined the effects of perceptual congruence in regard to pay justice or organizational justice in general. However, there are studies on congruence in other literatures that can be helpful to make predictions. Supervisor–employee congruence has been found to impact organizational outcomes, such as employee job performance, job satisfaction, and intention to stay in the organization (Wexley et al., 1980; Hatfield and Huseman, 1982; Turban and Jones, 1988; Moshavi et al., 2003; Ostroff et al., 2005; Bashshur et al., 2011; Tafvelin et al., 2017). The degree of agreement between supervisor and employee has been shown to be of such importance that it has been proposed that such congruence itself may be more important than the employees’ perception regarding the situation (Hasson et al., 2013). Previous research has found that the level of such congruence between how the supervisors evaluate themselves and the employees’ evaluations of them can influence employees’ attitudes and behaviors. This has been found in fields such as trust (Carter and Mossholder, 2015), psychological contracts (Kim et al., 2017), leadership (Atwater et al., 2009; Aarons et al., 2017; Carter et al., 2018), team interventions (Hasson et al., 2016), organizational learning (Tafvelin et al., 2017), and organizational support (Bashshur et al., 2011). Based on this, the following hypotheses were formulated:

*Hypothesis 3*. Employee levels of (a) job satisfaction, (b) organizational commitment, (c) intention to stay, and (d) job performance are higher when employees’ and supervisors’ pay justice ratings are similar and high, compared to similar and low.

*Hypothesis 4*. Employee levels of (a) job satisfaction, (b) organizational commitment, (c) intention to stay, and (d) job performance are higher when employees’ ratings of perceived pay justice are higher than their supervisors’ ratings of enacted pay justice, compared to when the supervisors’ ratings are higher than their employees.’

## Materials and Methods

### Setting

The data for the present study were collected from employees and supervisors employed in an industrial company that had been making considerable efforts to develop an individualized pay-setting system. The study sample was drawn from the population of all employees whose pay was regulated by the company’s pay-setting system, which was all of the supervisors and other white-collar employees at the company. Although the company is part of a multinational enterprise, our study only encompassed the Swedish sector of the company. The collective agreements between the employer federation and the unions did not specify pay raise figures or guarantee a certain minimum pay raise figure for its supervisors and other white-collar employees. Pay-setting supervisors had the discretion to grant pay raises that were much higher than the average pay raise to extremely high-performing employees and to withhold pay raises entirely in cases of very low performance (reductions in pay were not allowed).

### Sampling and Procedure

The data were collected using web-based surveys (via Qualtrics) administered in spring 2016, immediately after pay reviews had ended. The company communicated internally that they had decided to take part in a research project about individualized pay and that the company was interested in supervisors’ and employees’ perceptions about the pay system. The company also informed that invitations to participate in a questionnaire-based research investigation would be sent to all supervisors and employees from a research team. We sent out email invitations at the beginning of May 2016, in which the recipients were informed of the purpose of the project and that participation was voluntary; the invitations also explained that confidentiality was guaranteed and that no one outside of the project would have access to individual responses. The invitation emails each contained a personal link to one of the two versions of the questionnaire, depending on whether the recipient was a supervisor or employee. Four rounds of reminders were sent out, and the data collection concluded in June 2016. The research project was approved by the Regional Ethical Review Board in Stockholm (no. 2015/1733-31/5).

#### Supervisors

Out of the company’s 348 pay-setting supervisors, 213 chose to participate in the investigation, yielding a response rate of 61%. A non-response analysis between those who participated in the survey and those who did not was conducted using company records. There was no difference between the two groups in terms of age (*t*[df = 346] = 1.25, *p* = 0.212), tenure (*t*[df = 604] = 0.60, *p* = 0.546), or gender (χ^2^[df = 1] = 0.03, *p* = 0.861). A total of five multivariate outliers were detected using Mahalanobis distance and subsequently excluded, which resulted in a final sample of 208 participating supervisors. The mean age was 48 years, 26% were women, and the average employment tenure in the company as supervisor was 14 years.

#### Employees

A total of 1,191 of the company’s 2,793 non-supervisor, white-collar employees responded to the questionnaire, yielding a response rate of 43%. A non-response analysis between those who participated in the survey and those who did not was conducted using company records. There was no difference between the two groups in terms of age (*t*[df = 2614] = 1.74, *p* = 0.081), tenure (*t*[df = 2791] = −0.76, *p* = 0.449), or gender (χ^2^[df = 1] = 0.41, *p* = 0.521). A total of seven multivariate outliers were detected using Mahalanobis distance and subsequently excluded. In the next step, participating employees were matched with their supervisors using company records. In 566 instances, a match could be established, such that both supervisor and employee participated in the survey, had answered the justice questions, and were not categorized as outliers. An additional group comparison was conducted between those employees (*N* = 566) who were in the final employee sample and those employees who could not be matched with a participating supervisor. There was a significant difference between the two groups in tenure (*t*[df = 1188] = 2.42, *p* = 0.016), such that the final employee sample had somewhat longer tenure (M = 9.81 vs. M = 8.62). There was a significant difference between the two groups also in terms of justice enactment (*t*[df = 161] = 2.23, *p* = 0.027), such that supervisors of the final employee sample reported slightly higher justice enactment (M = 4.10 vs. M = 3.97). There were, however, no significant differences in age, gender, employees’ levels of justice perceptions, job satisfaction, organizational commitment, intention to stay, or job performance. The final employee sample (*N* = 566) had a mean age of 46 years, 34% were women, and the average employment tenure in the company was 10 years.

### Measures

#### Perceived Pay Justice

##### Employees

Employee justice perceptions in regard to pay setting were measured using an adapted version of a scale by Colquitt (2001). Specifically, we used a Swedish translation of Colquitt’s (2001) four-dimensional measure of perceived organizational justice that was translated to Swedish and adapted to a pay setting context by Andersson-Stråberg et al. (2007). During the translation process, all measures were translated into Swedish and then back-translated into English for verification by independent translators (Brislin, 1970). All of the items were measured on a five-point scale from 1 (to a very small extent) to 5 (to a very large extent). Distributive justice was measured by four items (e.g., “To what extent does your pay reflect the effort and dedication you have put into your work?”), for which the internal consistency (Cronbach’s alpha) was 0.94. Procedural justice was measured by six items (e.g., “To what extent have you been able to express your views and feelings on pay-setting issues?”), for which Cronbach’s alpha was 0.87. Informational justice was measured by five items (e.g., “To what extent has your boss explained the pay-setting process clearly and thoroughly?”), yielding a reliability estimate of 0.88. Interpersonal justice was measured by four items (e.g., “To what extent has your boss treated you with respect in relation to pay setting?”), with a reliability estimate of 0.86. The reliability of the entire scale of 20 items on pay justice was 0.95. Confirmatory factor analysis using Mplus (Muthén and Muthén, 1998) showed that a four-factor model that differentiated among distributive, procedural, informational, and interpersonal dimensions of justice (Colquitt, 2001) exhibited an acceptable fit to the data (χ^2^[df = 164] = 499.04, *p* < 0.001; RMSEA = 0.06; SRMR = 0.05; CFI = 0.95; TLI = 0.94). A second-order factor model, in which all four dimensions, in turn, were specified to load on a higher-order factor representing overall perceived justice in regard to pay setting, exhibited similar fit to the data (χ^2^[df = 166] = 512.82, *p* < 0.001; RMSEA = 0.06; SRMR = 0.05; CFI = 0.95; TLI = 0.94), which is in line with the presumption that overall justice can be a more parsimonious concept than including all four sub-dimensions (Ambrose et al., 2015). Taken together, these findings led us to treat justice perceptions as a unidimensional concept.

#### Enacted Pay Justice

##### Supervisors

The supervisors at the industrial company responded to justice-related questionnaire items based on the same scale (Colquitt, 2001), and following previous research adapting the Colquitt measure to reflect supervisor ratings of enacted justice (e.g., Zapata et al., 2013), the measure was adapted by us to address their enacted justice in regard to pay setting. The beginning of all of these items was thus changed to “To what extent do you as supervisor consider…,” and thus, the rest of one of the items measuring distributive justice was “…your employees’ pay to reflect the effort and dedication they have put into their work?” (Cronbach’s alpha = 0.80). Similarly, an example item measuring procedural justice ended with “…your employees to have had the opportunity to express their opinions and feelings on pay-setting issues?” (Cronbach’s alpha = 0.77), while items ending with “…that you have clearly and thoroughly explained the pay-setting process to your employees?” (Cronbach’s alpha = 0.81) and “…that you have treated your employees with respect in regard to pay setting?” (Cronbach’s alpha = 0.65) were among those measuring informational and interpersonal justice, respectively. The reliability (Cronbach’s alpha) for all 20 items was estimated to be 0.91. In this case as well, the results of confirmatory factor analysis showed that a four-factor model exhibited similar acceptable fit to the data (χ^2^[df = 164] = 318.02, *p* < 0.001; RMSEA = 0.07; SRMR = 0.07; CFI = 0.87; TLI = 0.85) compared to the second-order factor model with one underlying factor representing overall enacted justice (χ^2^[df = 166] = 321.66, *p* < 0.001; RMSEA = 0.07; SRMR = 0.07; CFI = 0.87; TLI = 0.85). Based on these findings, enacted justice was also considered to be a unidimensional rather than a four-dimensional phenomenon.

#### Work-Related Outcomes

##### Employees

All of the work-related attitudes and behaviors of interest were captured through items inquiring the employees’ degree of agreement or disagreement with certain statements, according to a Likert-type scale ranging from 1 (strongly disagree) to 5 (strongly agree). Job satisfaction was measured with three items (e.g., “I am satisfied with my job”) from Hellgren et al. (1999), which they based on Brayfield and Rothe (1951). The reliability (Cronbach’s alpha) was estimated to be 0.88. Organizational commitment was captured by four items measuring affective commitment, based on Allen and Meyer (1990), such as “I feel a strong sense of belonging to my organization” (Cronbach’s alpha = 0.86). Intention to stay was measured using the Sjöberg and Sverke (2000) three-item scale on turnover intention (e.g., “I am actively looking for other jobs”), which was then reverse-coded to capture intention to stay in the organization (Cronbach’s alpha = 0.86). Job performance was measured using a five-item scale (Hall and Hall, 1976) with statements such as “I strive for good quality in my work” (Cronbach’s alpha = 0.79).

### Analysis

Polynomial regression with response surface analysis (Edwards and Parry, 1993; Edwards, 1995, 2002; Shanock et al., 2010) was used to analyze the roles of employees’ perceived justice and supervisors’ perceptions of their enacted justice as well as the role of supervisor–employee congruence in regard to their perceptions of pay-setting justice. This approach enabled us to analyze the combined effects of employees’ perceived justice and supervisors’ enacted justice in pay setting on employees’ work-related attitudes and behavior. In line with recommendations by Shanock et al. (2010), three agreement groups were created: employees who reported higher values in perceived justice than their supervisors reported in enacted justice, employees who rated perceived justice equal to the ratings of their supervisors (within +/− 0.5 standard deviation), and employees who reported lower values in perceived justice than their supervisors reported in enacted justice. It is suggested that a considerable percentage (at least 10%) of disagreement needs to exist for further analysis to be meaningful.

Employee perceived justice ratings as well as supervisor enacted justice ratings were mean-centered before they were entered into the analysis. Separate analyses were conducted for each of the dependent variables (job satisfaction, organizational commitment, intention to stay, and job performance). The analyses included, in addition to the intercept (b_0_), the main effects of the employees’ ratings of perceived pay justice (b_1_) and of their respective supervisor’s ratings of enacted justice in regard to pay setting (b_2_), along with the square of the employees’ ratings (b_3_), the cross product of the employees’ and the supervisors’ ratings (b_4_), and the square of the supervisors’ ratings (b_5_). These analyses were used to address Hypothesis 1 (which predicted higher levels of employee perceived justice to have positive associations with work-related attitudes and behaviors) and Hypothesis 2 (which predicted higher levels of supervisor enacted justice to have positive associations with work-related attitudes and behaviors among employees). According to current analytical practice, response surface techniques can be utilized to interpret the results in cases where the variables in the polynomial regression explain a significant proportion of the variation in the dependent variable.

The analyses regarding Hypotheses 3 and 4, concerning levels of supervisor–employee (in) congruence, included the two quadratic terms, as well as the interaction between supervisors’ and employees’ ratings, which made the response surface technique the most direct way of testing these hypotheses. We analyzed the role of congruence by calculating the slope (a_1_) and the curvature (a_2_) for the line representing the congruence between employees’ and supervisors’ ratings. Similarly, the slope (a_3_) and the curvature (a_4_) for the line representing a lack of congruence between employees’ and supervisors’ ratings were also calculated. The slope represents how congruence/incongruence between the two predictors relates to a dependent variable, while the curvature illustrates whether the association between (in)congruence and the dependent variable is linear or non-linear, that is, in what way the dependent variable is affected if the supervisor or employee under- or overestimates justice compared to the other party. This method makes it possible to graphically illustrate the shape of the surface between the two lines (representing congruence and lack of congruence).

## Results

Table 1 presents the means, standard deviations, reliability estimates (Cronbach’s alpha), and intercorrelations for the variables under study. The mean level of the supervisors’ ratings of enacted pay justice was considerably higher than the employees’ mean level of perceived justice (4.08 and 3.24, respectively). The mean levels of employees’ ratings of work-related attitudes and behavior were relatively high overall, especially for job performance, which indicated a restriction of range, with a mean value of 4.45 and a low standard deviation (0.48). The employees’ ratings of perceived justice in regard to pay setting were positively associated with all four dependent variables, while the supervisors’ ratings of their enacted justice were not significantly associated with any of the employee outcomes. Perceived and enacted justice were not found to be associated with each other. Correlations between the dependent variables were generally high, with the exception of a weak correlation (0.19) between intention to stay and job performance.

**TABLE 1:** Descriptive statistics, reliability estimates, and correlations for the study variables.

Variable	1	2	3	4	5	6
1. Perceived pay justice (employee)						
2. Enacted pay justice (supervisor)	0.00					
3. Job satisfaction	0.37***	0.01				
4. Organizational commitment	0.38***	–0.03	0.70***			
5. Intention to stay	0.37***	0.00	0.65***	0.52***		
6. Job performance	0.10*	0.04	0.45***	0.50***	0.19***	
Mean	3.35	4.10	3.89	3.84	3.98	4.45
Standard deviation	0.80	0.46	0.86	0.79	1.10	0.48
Cronbach’s alpha	0.95	0.91	0.88	0.86	0.86	0.79

***p < 0.001, **p < 0.01, *p < 0.05 (N = 566).

Calculating the percentages of agreement groups revealed that 26.5% of the employees were in agreement with their supervisors. A total of 37.1% of employees reported higher justice perceptions than their supervisors reported justice enactment, and the remaining 36.4% were classified into the other disagreement group, such that supervisors reported higher justice enactment than their employees reported justice perceptions. This analysis confirmed that disagreement was sufficiently large to warrant further analyses of the consequences of the differences between employee and supervisor (see Shanock et al., 2010).

Table 2 presents the results of the polynomial regression analyses. In total, the predictors explained significant proportions of the variances in job satisfaction (14%), organizational commitment (14%), and intention to stay (15%)—as well as in job performance, although the proportion of explained variance was low (3%). According to the unstandardized (*B*) and standardized (β) regression coefficients, employees’ perceived pay justice was positively associated with all of the outcomes, thus supporting Hypothesis 1. Hypothesis 2, however, was not supported, as supervisors’ enacted justice was not found to be significantly associated with any of the dependent variables.

**TABLE 2:** Results of the polynomial regression analysis, including response surface analysis, to predict work-related outcomes.

Variable	Job satisfaction		Organizational commitment		Intention to stay		Job performance	
Polynomial regression results	B (SE)	β	B (SE)	β	B (SE)	β	B (SE)	β
Constant (b_0_)	3.88 (0.05)		3.86 (0.05)		4.09 (0.07)		4.41 (0.03)	
Perceived justice (b_1_)	0.40 (0.04)	0.37***	0.37 (0.04)	0.38***	0.47 (0.06)	0.35***	0.08 (0.03)	0.13**
Enacted justice (b_2_)	0.02 (0.08)	0.01	−0.06(0.07)	–0.04	−0.02(0.10)	–0.01	0.04 (0.05)	0.04
Perceived justice squared (b_3_)	0.01 (0.04)	0.01	0.00 (0.04)	0.00	−0.13(0.05)	−0.10**	0.07 (0.03)	0.13**
Perceived * enacted (b_4_)	0.04 (0.10)	0.02	0.01 (0.09)	0.01	0.24 (0.12)	0.08^#^	0.00 (0.06)	0.00
Enacted justice squared (b_5_)	0.03 (0.14)	0.01	−0.12(0.12)	–0.04	−0.12(0.17)	–0.03	−0.03(0.08)	–0.01
*R*^2^	0.14***		0.14***		0.15***		0.03*	

**Surface tests**	**B (SE)**	**t**	**B (SE)**	**t**	**B (SE)**	**t**	**B (SE)**	**t**

**Congruence line**								
Slope (a_1_ = b_1_ + b_2_)	0.42 (0.09)	4.67***	0.31 (0.08)	3.83***	0.45 (0.11)	3.99***	0.12 (0.05)	2.26*
Curvature (a_2_ = b_3_ + b_4_ + b_5_)	0.08 (0.17)	0.47	−0.11(0.15)	–0.69	−0.01(0.21)	–0.05	0.05 (0.10)	0.46
**Incongruence line**								
Slope (a_3_ = b_1_ – b_2_)	0.38 (0.09)	4.27***	0.43 (0.08)	5.36***	0.50 (0.11)	4.39***	0.04 (0.05)	0.73
Curvature (a_4_ = b_3_ – b_4_ + b_5_)	0.00 (0.17)	0.01	−0.01(0.15)	–0.86	−0.49(0.21)	−2.32*	0.04 (0.10)	0.45

B = unstandardized regression coefficient; SE = standard error; β = standardized regression coefficient. ***p < 0.001, **p < 0.01, *p < 0.05, # p < 0.10 (N = 566).

**FIGURE 2:**
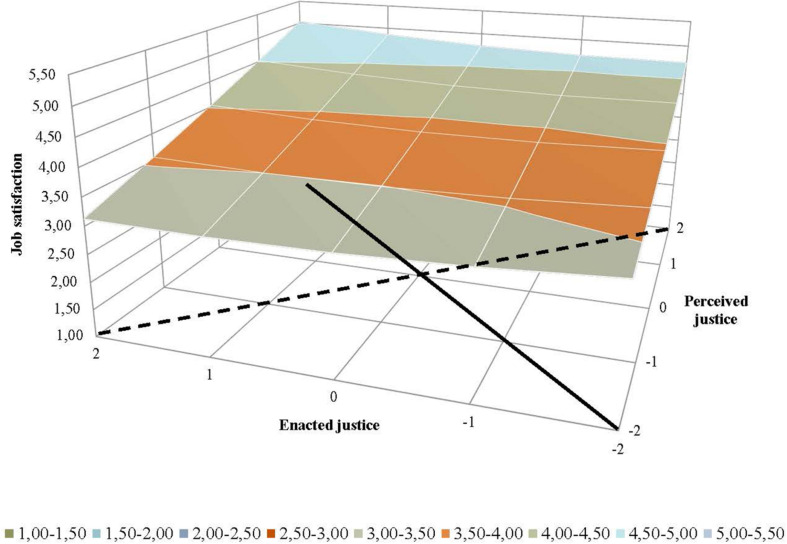
Effects of (in)congruence between employees’ perceived pay justice and supervisors’ enacted pay justice on employees’ job satisfaction.

**FIGURE 3:**
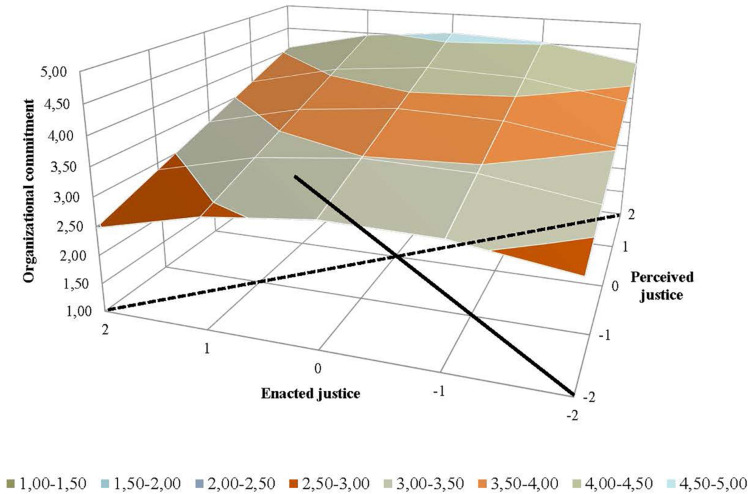
Effects of (in)congruence between employees’ perceived pay justice and supervisors’ enacted pay justice on employees’ organizational commitment.

**FIGURE 4:**
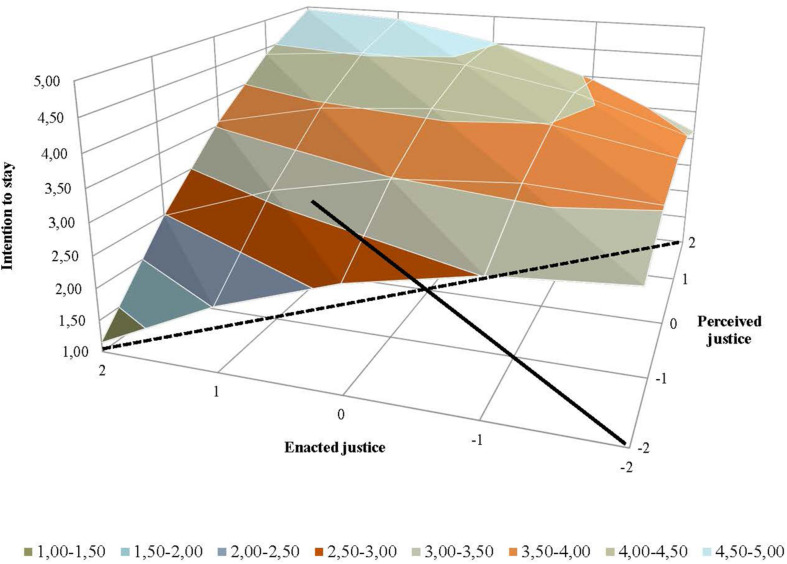
Effects of (in)congruence between employees’ perceived pay justice and supervisors’ enacted pay justice on employees’ intention to stay.

**FIGURE 5:**
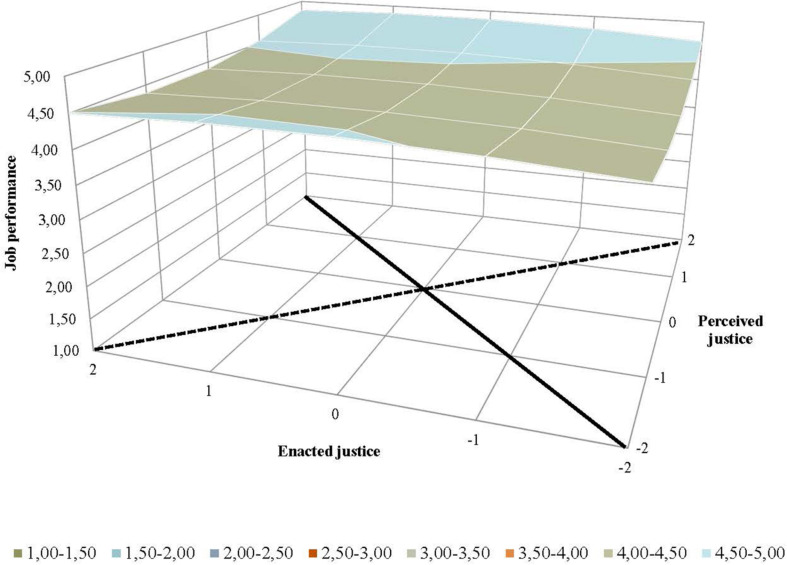
Effects of (in)congruence between employees’ perceived pay justice and supervisors’ enacted pay justice on employees’ job performance.

Table 2 also shows the results from the response surface analyses, which were conducted to analyze the effects of the extent of supervisor–employee congruence (Hypotheses 3 and 4). Graphic representations of the results from the response surface analyses were generated to facilitate interpreting how the relationship between employees’ perceived justice and supervisors’ ratings of enacted justice related to employees’ job satisfaction (Figure 2), organizational commitment (Figure 3), intention to stay (Figure 4), and job performance (Figure 5). For both perceived and enacted justice, the values are presented at up to 2 standard deviations (SD) above and below the centered means. In these figures, the solid line represents congruence, and the dashed line represents incongruence, between the employees’ perceived justice ratings and the supervisors’ enacted justice ratings in relation to the outcome variables.

With regard to job satisfaction, the slope was found to be positive for the congruence line (a_1_) and the incongruence line (a_3_), while the coefficients for the curvatures of these lines (a_2_ and a_4_, respectively) were not significant (see Table 2). As Figure 2 shows, the extent of congruence between supervisors’ enacted pay justice ratings and employees’ ratings of perceived pay justice related to higher levels of employee job satisfaction. More specifically, the mean values for job satisfaction were highest when the ratings of employees’ perceived justice and supervisors’ enacted justice were both high (as indicated in the back left corner of Figure 1, where the solid congruence line ends). The mean values for this outcome were lowest when both parties had low ratings for these measures (as indicated in the front right corner, where the solid line begins). These results support Hypothesis 3a. As the dashed line representing lack of congruence shows, the mean values for job satisfaction were generally higher when the employees’ ratings of perceived pay justice were higher than the supervisors’ ratings of enacted justice (as indicated in the back right part) compared to when the supervisors rated their enacted justice higher than the employees’ perceived justice (as indicated in the front left part). These results also support Hypothesis 4a. The back section of Figure 1, indicating the main effect of employees’ perceived justice in regard to pay setting, shows that the highest levels of job satisfaction occurred among employees who perceived high levels of justice irrespective of their supervisors’ enacted justice ratings.

Regarding organizational commitment, the slopes were positive for both the congruence line and the incongruence line (a_1_ and a_3_, respectively, in Table 2), while the coefficients for curvature around these lines (a_2_ and a_4_, respectively) were non-significant. Figure 3 shows that the mean values for organizational commitment were higher when the employees’ and supervisors’ pay justice ratings were high (indicated in the back left corner) than when these ratings were low (indicated in the closer part of the solid line), which is in line with Hypothesis 3b’s prediction. The levels of organizational commitment were highest when the employees rated perceived justice in regard to pay setting higher than their supervisors rated enacted justice (indicated in the right part of the dashed line of incongruence), while the lowest levels occurred when the supervisors rated enacted justice as high in conjunction with their employees rating perceived justice as low (indicated in the left part of the dashed line). These results support Hypothesis 4b.

A somewhat different pattern emerged with regard to employees’ intention to stay in the organization. More specifically, as in previous analyses, the slope was significant for the congruence line (a_1_) as well as for the incongruence line (a_3_), but here the curvature for the incongruence line (a_4_) was also found to be significant (see Table 2). The results regarding intention to stay provide support for the general presumption that a higher level of congruence is associated with more positive work-related outcomes among employees. As can be seen in Figure 4, the level of intention to stay was higher when employees’ and their supervisors’ ratings of pay-setting justice were both high, while the level was much lower when these ratings were both low (indicated by the solid line), which supports Hypothesis 3c. As illustrated by the dashed, incongruence line, intention to stay was lowest among the employees whose perceived justice ratings were low while their supervisors’ enacted justice ratings were very high. A comparatively higher level of intention to stay was found among employees who rated their perceived justice more highly than their supervisor’s rated enacted justice. The curvature indicates that the more the ratings differed—in terms of supervisors’ ratings of enacted justice being higher than their employees’ ratings of perceived justice—the lower the employees’ intention to stay was. This supports Hypothesis 4c.

When it comes to job performance, only the slope for the congruence line (a_1_) was found to be significant (see Table 2). The mean values in this analysis were generally high regardless of the employees’ perceived justice or the supervisors’ enacted justice ratings. The weak positive slope for congruence (the solid line in Figure 5) illustrates that the mean values for job performance were higher when the employees’ and supervisors’ ratings of pay-setting justice were both high as compared to when these ratings were lower. Although the differences in mean values were marginal, these results provide some degree of support for Hypothesis 3d. No support, however, was found for Hypothesis 4d, as there were no differences along the dashed line representing incongruence between employees’ and supervisors’ ratings of justice in regard to pay setting. The generally high mean values for job performance, as shown in the back part of the figure, represent the main effect of employees’ perceived justice. Overall, the analysis provides only limited support for the general assumption that congruence between employees and supervisors in regard to justice perceptions would be associated with higher job performance.

## Discussion

The aim of this study was threefold and included investigating: (1) the impact of employees’ perceived pay justice on their levels of job satisfaction, organizational commitment, intention to stay, and job performance; (2) the effects of supervisors’ enacted pay justice on these outcomes; and (3) whether a congruence between employees’ perceived pay justice and supervisors’ enacted pay justice can affect these outcomes. One striking characteristic of the present study is that it takes into account both employees’ and supervisors’ perceptions of justice in relation to pay setting. Another distinguishing feature of our study is that it, in line with several studies (Ambrose and Schminke, 2009; Koopman et al., 2019), examines fairness perceptions in terms of overall justice rather than focusing on various justice dimensions. The present study also contributes to the state of knowledge in this area by being the first study, to the best of our knowledge, to examine the effects of there being perceptual congruence between employees’ perceived justice and supervisors’ enacted justice in regard to pay setting.

### Effects of Perceived Pay Justice

The first objective of the present study was to examine the potential effects of employees’ perceptions of justice in regard to pay setting on their work-related attitudes and behaviors. The results showed that higher levels of perceived pay justice were positively associated with all of the investigated work-related attitudes and behaviors, which supports Hypothesis 1a–d. The results are in line with previous findings, both in regard to general organizational justice (Colquitt et al., 2001, 2013; Rupp et al., 2014) and pay justice (Tekleab et al., 2005; Andersson-Stråberg et al., 2007). Also consistent with previous research (Colquitt et al., 2013) is our finding that employee justice perceptions were more strongly associated with work-related attitudes than with work-related behavior, such as job performance. Studies on organizational justice have found that a high level of perceived justice is associated with higher job satisfaction, job performance, intention to stay, and organizational commitment (Colquitt et al., 2001, 2013; Rupp et al., 2014), and although pay justice has not been studied to the same extent, there are also indications that it is, similarly, related to these outcomes (Tekleab et al., 2005; Andersson-Stråberg et al., 2007). For the literature on overall justice (Ambrose et al., 2015), this study is a step forward to considering overall justice as fairness perceptions in the context of pay setting. This is exactly what Colquitt and Rodell (2015) have suggested: to use overall justice in specific contexts.

While this support of the hypothesis adds to the literature on overall justice in pay setting, it is of even more practical importance. That employees perceive a high degree of pay justice can thus be seen as crucial. It matters not only that pay raises are perceived as fair by employees but also that the pay setting in general—in terms of the procedures for determining pay raises, information regarding the pay system, and employee–employer interaction in relation to pay setting—is viewed as fair (Tekleab et al., 2005; Andersson-Stråberg et al., 2007). If employees perceive that their pay setting has been fair, they will believe that the supervisor has good intentions toward them, even if they received a lower pay raise than they had been expecting (Greenberg, 2004). If the procedures for pay setting are perceived as just, the likelihood for pay raises being perceived as just increases. This is in line with social exchange theory and the notion that employees who perceive that they are treated fairly will reciprocate with favorable work-related attitudes and behavior (Cropanzano and Mitchell, 2005). A qualitative study with HR directors in the US has found that the perceived fairness of criteria in performance appraisals is positively associated with company performance (Kim, 2016). This finding highlights that fairness of pay-setting systems may even have wider benefits for the whole organization. The present study, therefore, illustrates that it is important for organizations to intensively work to develop their pay-setting processes in order to increase the likelihood that their employees perceive a high level of pay justice and thus ultimately contribute to fulfilling the desired positive outcomes of the pay system.

### Effects of Enacted Pay Justice

The second aim of our study was to investigate if supervisors’ ratings of enacted justice in pay setting relate to their employees’ work-related attitudes and behaviors. Since the results revealed that the degree of enacted pay justice among supervisors was unrelated to employee attitudes and behaviors, no support was found for Hypothesis 2a–d. To our knowledge, no previous study has investigated whether supervisors’ enacted pay justice is associated with employees’ attitudes and behavior. The present results suggest that employees’ attitudes and behaviors are shaped by factors other than the supervisor’s enacted justice in regard to pay setting (i.e., insofar as the ratings of enacted justice reflect their actual behavior in relation to pay setting). Even though the supervisors’ behaviors have previously been found to impact employees’ attitudes and behavior (Karam et al., 2019), the present results are in line with studies indicating that employees do not passively receive justice from their supervisors (Zapata et al., 2013; Huang et al., 2017; Karam et al., 2019). Along with supervisors having been identified as having a key role in employees’ justice perceptions, it has also been pointed out that supervisors’ enacted justice can act as an antecedent to employees’ perceived justice (Koopman et al., 2019) and that employees shape their justice perceptions based on their supervisors’ actions. The subsequent effects on employees’ attitudes and behaviors could thus be related to the employees’ perceived justice rather than to the supervisor’s enacted justice.

However, even on a correlational level, no significant relationship was found in our study between the perceived justice ratings of the employees and the enacted justice ratings of the supervisors. In the few previous studies on justice that have measured both perceived and enacted justice, however, weak but significant correlations have been found between the two (Zapata et al., 2013; Huang et al., 2017; Koopman et al., 2019). For instance, Koopman et al. (2019) found a 0.33 correlation between overall justice perceptions and overall justice enactment. There are several possible explanations for why our study did not reveal a positive correlation between perceived and enacted justice. One concerns the fact that the current study examined overall justice, thereby averaging justice dimensions. While this has also been done in other studies (e.g., Koopman et al., 2019), it should be noted, however, that Koopman and colleagues asked the employees to rate how just they perceived their supervisor to be in general and the supervisors how just they perceived themselves to be in general, not in regard to a specific context. In contrast, our ratings capture perceived and enacted justice in relation to pay setting. Another explanation relates to the measurement of justice enactment. Like in previous studies (e.g., Koopman et al., 2019), each supervisor was asked to indicate how he or she generally acts in relation to all his or her subordinates, and it could be that supervisors were affected by social desirability when making judgments about their own behavior. However, it is not difficult to imagine that participants in the previous studies on the topic of perceived and enacted justice would also have been prone to social desirability. Furthermore, it might also be the case that enacted justice impacts outcomes over time, potentially having more wide-ranging effects for the organization, for example, in regard to strengthening future leadership or recognizing aspects of the pay system that are not advantageous to promoting justice and, in turn, its positive outcomes. One important implication of these results and speculations is that it is surprising how little empirical work has been done on the association between justice enactment and employee outcomes. As stated previously, justice scholars assume that there is a positive and substantial association between the two; yet, what this association actually looks like in terms of strength, measure of justice, and potential moderators or mediators lies in the dark. We urge researchers to target this blind spot in the literature.

### Effects of Perceptual Congruence

The third objective of the study was to investigate the effects of perceptual (in)congruence in regard to pay setting on employee attitudes and behaviors. When the ratings of supervisors and employees were congruent in respect to perceiving high levels of pay justice, it was found to be associated with higher levels of work-related attitudes and behaviors among employees. This finding, which supports Hypothesis 3a–d, is in line with previous research indicating that a congruence of higher values is, to a greater extent, associated with positive outcomes as compared to a congruence of low values (Bashshur et al., 2011). The effects of pay-justice congruence have not been studied previously, but studies of congruence in other organizational contexts have shown that it may be of importance for employee outcomes, whether such congruence concerns trust (Carter and Mossholder, 2015), values (Ostroff et al., 2005), psychological contract (Kim et al., 2017), power distance (Cole et al., 2013), leadership (Atwater et al., 2009; Aarons et al., 2017; Carter et al., 2018), team interventions (Hasson et al., 2016), communication (Hatfield and Huseman, 1982; Benlian, 2014), organizational learning (Tafvelin et al., 2017), organizational support (Bashshur et al., 2011), goal accomplishment (Gibson et al., 2009), or performance evaluations (Wexley et al., 1980).

In line with this, we found that congruence between supervisors and employees in regard to higher levels of pay justice was associated with more favorable work-related attitudes and behaviors among the employees. In regard to Hypothesis 3, congruence between how the supervisor and the employee perceived the level of justice in connection to pay setting seems to clearly have an impact on employee attitudes and behaviors. More specifically, when supervisors and employees were in agreement on the level of justice—and especially when they were in agreement that the level of pay-related justice was high—we found such congruence to be related to higher levels of work-related attitudes and behavior (Hypothesis 3a–d). In contrast, when supervisors’ and employees’ ratings were incongruent, that is, when the supervisors’ enacted pay-justice ratings were higher than the employees’ perceived pay-justice ratings, it was related to lower levels of employee job satisfaction, organizational commitment, and intention to stay, which is in line with Hypothesis 4a–c, while this was not true for job performance (Hypothesis 4d). However, when employees’ pay-justice ratings were higher than the supervisors’ enacted pay justice ratings, it was found to be associated with higher levels of employee job satisfaction, organizational commitment, and intention to stay. The results addressing Hypothesis 4a–c are in line with results from previous research, which have shown that when supervisors’ ratings are higher than the employees’ ratings, they are related to less positive outcomes (Yammarino and Atwater, 1993; Atwater et al., 1998; Bashshur et al., 2011; Tafvelin et al., 2017).

Blau (1964) distinguished between social and economic exchanges in his social exchange theory. Individualized pay setting is more than just an economic exchange; it is also a social exchange. More specifically, the measure of pay justice in the present study (based on Colquitt, 2001) illustrates that pay justice represents much more than just one’s reaction to an economic exchange (e.g., a pay raise, or lack thereof), as it also takes into account the perceived fairness of the entire process as well as whether the supervisor treats employees fairly and provides information in a timely manner. While supervisors’ enacted justice was not found to, on its own, predict employee attitudes and behaviors, supervisor–employee congruence regarding high levels of justice was associated with more positive employee attitudes and behavior. This suggests that when there is a shared positive view of pay justice between supervisor and employee, it will have positive effects for the employee. To have employees who are satisfied, are committed, wish to remain with the organization, and perform well is also likely to benefit organizations.

Within the pay-setting context, a higher degree of congruence may be an indicator that a fruitful two-way communication has taken place and, more specifically, that the employee was able to gather insight into the supervisor’s approach and, thus, a better understanding of the supervisor’s actions and pay decisions. Congruence suggests that there is a functioning exchange between the supervisor and the employee. The present study focused on employees and their immediate supervisors, but congruence regarding pay justice does not occur in a vacuum, and future studies could investigate the broader conditions that promote stronger congruence. Practically, the results of this study highlight that supervisors need to take into consideration the justice perceptions of employees in order to guarantee positive employee outcomes. Another implication is that to better explain employee outcomes, it may be worthwhile to include not just employees’ perceptions but also indicators of the interaction to their immediate supervisor. The next natural step is to identify what factors may predict congruence in regard to pay justice perceptions. In studies on the agreement between supervisors and employees about their relationship quality, congruence was more likely with higher interaction frequency, longer relationship length, and higher perceived effort from both sides (Maslyn and Uhl-Bien, 2001; Tekleab and Taylor, 2003; Sin et al., 2009). Similar concepts also seem relevant for congruence on organizational justice and could be investigated in future studies (Fleenor et al., 2010).

### Methodological Considerations

The cross-sectional nature of this study does not allow conclusions to be drawn about the direction of the associations (Bollen, 1989). In the future, however, it would be interesting to investigate, using a quasi-experimental design with pre-tests and post-tests, how a change of pay system relates to justice perceptions and employee–supervisor congruence. Although this study did not aim to identify causal relationships, it may be valuable for future research to examine how employees’ justice perceptions in regard to pay setting develop over time, especially since individualized pay setting is often an annually occurring process (Andersson-Stråberg et al., 2007). For instance, an interesting approach could be to investigate the process more closely by investigating employee justice perceptions and expectations in regard to the pay discussion process at several junctures, such as shortly before and after the discussion. Another inquiry a few weeks afterward may be particularly revealing with regard to altered justice perceptions.

Another limitation of the study has to with the self-reported nature of the data, which can involve a risk of common method variance (CMV). It has been suggested that CMV can distort associations due to adding systematic variance (Podsakoff et al., 2003). However, prominent researchers have cast doubts about the importance of CMV in organizational research (Spector, 2006). Moreover, it has been noted that method bias “cannot inflate (but does deflate) quadratic and interaction effects” (Podsakoff et al., 2012, p. 564). Hence, the potential risk here is, rather, that some associations were underestimated. In addition, we used data from two different sources—employees and supervisors. To nevertheless reduce the potential risk of CMV, we followed a number of procedural recommendations from Podsakoff and colleagues (e.g., using negatively coded items and different response formats for scales, physically spreading out independent and dependent variables in the questionnaires, and reminding participants about their confidentiality).

In the present study, the participants were white-collar employees from a private-sector company in Sweden. The industrial relations system in Sweden is regulated by collective agreements, with unions typically having a strong influence. Regarding pay setting, pay raises are generally relatively modest in Sweden, and historically, there has been an agreed-upon norm that pay across jobs should not vary too much. The Swedish context and the circumstance that the study took place in a company with a highly developed pay system might have limited the generalizability of the results. Other studies are needed to investigate the effects of justice perceptions and of employee–supervisor congruence in other countries and in other organizations with different pay systems. To test the generalizability of the present findings, the study should therefore be replicated in other contexts, such as in companies that have not intensively focused on developing a pay-setting system, among other occupational groups, or in other countries. However, an advantage of all study participants being employed in the same organization is that this makes it more likely that the results are due to justice perceptions per se and not due to differences between pay systems.

Another potential limitation is that the employees rated how just their particular supervisor was, while the supervisors rated how just they were toward their employees on average. As supervisors had up to 24 employees each, it was not practical to ask them to fill out a separate questionnaire for each employee. This approach has also been utilized in previous studies (e.g., Koopman et al., 2019). Another option that has been used in other studies (e.g., Hasson et al., 2016) is for the supervisor to select one or a few employees and to have the relevant responses concern interactions with those individuals. With such an approach, even though it is advantageous to capture a supervisor’s ratings in regard to a particular employee, there is a risk that the supervisor will only select employees with whom they have had positive interactions, which can bias the results.

Moreover, there may have been a self-selection bias, in that only those with a good mutual relationship decided to participate in the study. In line with recommendations by Shanock et al. (2010), there was sufficient disagreement between participating employees and supervisors to warrant further analyses. Also, the information potential participants in the organization received included general information regarding the interest of the project to study perceptions with regard to the pay-setting process in the company, with a guarantee for full confidentiality. In addition, we conducted several non-response analyses, which provided limited evidence that self-selection may be a problem. However, it is impossible to test whether the supervisors who participated in the study had better relations with their subordinates than those who did not respond to the survey or whether (in)congruence differed between the effective sample and those participants who could not be matched to their supervisors.

Yet another potential limitation is that all analyses were conducted without controlling for confounders. However, supplementary analyses with covariates (age, gender, and tenure from company records) provided very similar results (not reported). While the intercepts were slightly lower in the analyses controlling for these confounders, the covariates generally did not predict any of the outcomes—with one exception (age was had a significant, positive association with intention to stay). Most importantly, the regression weights remained more or less identical, and no variable central to the polynomial analysis became non-significant or significant compared to the analyses without covariates. The same was found for the surface tests, which remained more or less identical. Again, no effect became significant or non-significant as compared to the results not controlling for these potential confounders. In addition, the plots from the surface tests remained more or less identical.

## Conclusion

To reward employees in a way that is commensurate with their performance is easier said than done. Individualized pay setting involves more than just an economic exchange in the form of a pay raise. The present study illustrates not only the importance of employees perceiving that their pay setting is just but also that it is important for the perceptions of justice to be high and similar between supervisor and employee. This is one of the first studies to investigate the congruence between employees’ justice perception ratings and supervisors’ justice enactment ratings in the context of pay setting. The effects were found to be especially positive when the congruence reflected high levels of perceived pay-setting justice, while the most negative effects were found when supervisors rated their enacted pay justice higher than their employees rated their perceived pay justice. While individualized pay setting is expected to lead to positive outcomes in the forms of improved work-related attitudes and behaviors among employees, the present study confirms the important role of perceived justice in regard to pay setting and also emphasizes the importance of supervisors’ actions in regard to pay-setting justice. More specifically, it highlights that congruence between supervisors’ and employees’ ratings of justice in relation to pay setting are important for employees’ work-related attitudes and behavior.

## Data Availability Statement

The datasets generated for this study will not be made publicly available. The ethical approval does not allow the data to be shared outside the research group.

## Ethics Statement

The research project was approved by the Regional Ethical Review Board in Stockholm (no. 2015/1733-31/5). Written informed consent for participation was not required for this study in accordance with the national legislation and the institutional requirements.

## Author Contributions

SM provided the idea for the study. All authors participated in the conceptualization as well as the design and analytical approach of the study. MS, CE, and SM carried out the statistical analyses. SM wrote the first draft of the manuscript, primarily, CE, MS, and HF contributed to the revisions and writing of the final manuscript. All authors approved the final version and agreed to be accountable for all aspects of the work.

## Conflict of Interest

The authors declare that the research was conducted in the absence of any commercial or financial relationships that could be construed as a potential conflict of interest.

## References

[B1] Aarons G. A. Ehrhart M. G. Torres E. M. Finn N. K. Beidas R. S. (2017). The humble leader: association of discrepancies in leader and follower ratings of implementation leadership with organizational climate in mental health. *Psychiatr. Serv.* 68 115–122. 10.1176/appi.ps.201600062 27691380 PMC5462527

[B2] Adams J. S. (1965). “Inequity in social exchange,” in *Advances in Experimental Social Psychology*, ed. Berkowitz L. (New York, NY: Academic Press), 267–289.

[B3] Allen N. J. Meyer J. P. (1990). The measurement and antecedents of affective, continuance and normative commitment to the organization. *J. Occup. Psychol.* 63 1–18. 10.1111/j.2044-8325.1990.tb00506.x

[B4] Ambrose M. L. Schminke M. (2009). The role of overall justice judgments in organizational justice research: a test of mediation. *J. Appl. Psychol.* 94 491–500. 10.1037/a0013203 19271803

[B5] Ambrose M. L. Wo D. X. H. Griffith M. D. (2015). “Overall justice: past, present, and future,” in *The Oxford Handbook of Justice in the Workplace*, eds Cropanzano R. Ambrose M. L. (New York, NY: Oxford University Press), 109–135.

[B6] Andersson-Stråberg T. Sverke M. Hellgren J. (2007). Perceptions of justice in connection with individualized pay setting. *Econ. Ind. Democ.* 28 431–464. 10.1177/0143831x07079356

[B7] Atwater L. Wang M. Smither J. W. Fleenor J. W. (2009). Are cultural characteristics associated with the relationship between self and Others’, ratings of leadership? *J. Appl. Psychol.* 94 876–886. 10.1037/a0014561 19594231

[B8] Atwater L. E. Ostroff C. Yammarino F. J. Fleenor J. W. (1998). Self–other agreement: Does it really matter? *Pers. Psychol.* 51 577–598. 10.1111/j.1744-6570.1998.tb00252.x

[B9] Bashshur M. R. Hernandez A. Gonzalez-Roma V. (2011). When managers and their teams disagree: a longitudinal look at the consequences of differences in perceptions of organizational support. *J. Appl. Psychol.* 96 558–573. 10.1037/a0022675 21341884

[B10] Beer M. Cannon M. D. (2004). Promise and peril in implementing pay-for-performance. *Hum. Resour. Manage.* 43 3–20. 10.1002/hrm.20001

[B11] Benlian A. (2014). Are we aligned…Enough? The effects of perceptual congruence between service teams and their leaders on team performance. *J. Serv. Res.* 17 212–228. 10.1177/1094670513516673

[B12] Bies R. J. Moag J. F. (1986). “Interactional justice: communication criteria of fairness,” in *Research on Negotiations in Organizations*, eds Lewicki R. J. Sheppard B. H. Bazerman M. H. (Greenwich, CT: JAI Press).

[B13] Blau P. M. (1964). *Exchange and Power in Social Life.* New York: Wiley.

[B14] Bollen K. A. (1989). *Structural Equations with Latent Variables.* New York, NJ: John Wiley & Sons, Inc.

[B15] Brayfield A. Rothe H. F. (1951). An index of job satisfaction. *J. Appl. Psychol.* 35 307–311. 10.1037/h0055617

[B16] Brebels L. De Cremer D. Van Dijke M. Van Hiel A. (2011). Fairness as social responsibility: a moral self-regulation account of procedural justice enactment. *Br. J. Manage.* 22 S47–S58. 10.1111/j.1467-8551.2010.00715.x

[B17] Brislin R. W. (1970). Back-translation for cross-cultural research. *J. Cross Cult. Psychol.* 1 185–216. 10.1177/135910457000100301

[B18] Carter M. Z. Mossholder K. W. (2015). Are we on the same page? The performance effects of congruence between supervisor and group trust. *J. Appl. Psychol.* 100 1349–1363. 10.1037/a0038798 25688640

[B19] Carter M. Z. Mossholder K. W. Harris J. N. (2018). Congruence effects of contingent reward leadership intended and experienced on team effectiveness: the mediating role of distributive justice climate. *J. Occup. Organ. Psychol.* 91 465–485. 10.1111/joop.12210

[B20] Choi J. Chen C. C. (2007). The relationships of distributive justice and compensation system fairness to employee attitudes in international joint ventures. *J. Organ. Behav.* 28 687–703. 10.1002/job.438

[B21] Cohen-Charash Y. Spector P. E. (2001). The role of justice in organizations: a meta-analysis. *Organ. Behav. Hum. Decis. Process.* 86 278–321. 10.1006/obhd.2001.2958

[B22] Cole M. S. Carter M. Z. Zhang Z. (2013). Leader-team congruence in power distance values and team effectiveness: the mediating role of procedural justice climate. *J. Appl. Psychol.* 98 962–973. 10.1037/a0034269 24060159

[B23] Colquitt J. A. (2001). On the dimensionality of organizational justice: a construct validation of a measure. *J. Appl. Psychol.* 86 386–400. 10.1037/0021-9010.86.3.386 11419799

[B24] Colquitt J. A. Conlon D. E. Wesson M. J. Porter C. O. L. H. Ng K. Y. (2001). Justice at the millenium: a meta-analytic review of 25 years of organizational justice research. *J. Appl. Psychol.* 86 425–445. 10.1037/0021-9010.86.3.425 11419803

[B25] Colquitt J. A. Rodell J. B. (2015). “Measuring justice and fairness,” in *The Oxford Handbook of Justice in the Workplace*, eds Cropanzano R. Ambrose M. L. (Oxford: Oxford University Press), 187–202.

[B26] Colquitt J. A. Scott B. A. Rodell J. B. Long D. M. Zapata C. P. Conlon D. E. (2013). Justice at the millennium, a decade later: a meta-analytic test of social exchange and affect-based perspectives. *J. Appl. Psychol.* 98 199–236. 10.1037/a0031757 23458336

[B27] Cropanzano R. Byrne Z. S. Bobocel D. R. Rupp D. E. (2001). Moral virtues, fairness heuristics, social entities, and other denizens of organizational justice. *J. Vocat. Behav.* 58 164–209. 10.1006/jvbe.2001.1791

[B28] Cropanzano R. Greenberg J. (1997). “Progress in organizational justice: tunneling through the maze,” in *International Review of Industrial and Organizational Psychology*, eds Cooper C. L. Robertson I. T. (New York, NY: John Wiley & Sons), 317–372.

[B29] Cropanzano R. Mitchell M. S. (2005). Social exchange theory: an interdisciplinary review. *J. Manage.* 31 874–900. 10.1177/0149206305279602

[B30] Deutsch M. (1975). Equity, equality, and need: What determines which value will be used as the basis of distributive justice? *J. Soc. Issues* 31 137–149. 10.1111/j.1540-4560.1975.tb01000.x

[B31] Edwards J. R. (1995). Alternatives to difference scores as dependent variables in the study of congruence in organizational research. *Organ. Behav. Hum. Decis. Process.* 64 307–324. 10.1006/obhd.1995.1108

[B32] Edwards J. R. (2002). “Alternatives to difference scores: polynomial regression analysis and response surface methodology,” in *Measuring and Analyzing Behavior in Organizations: Advances in Measurement and Data Analysis*, eds Drasgow F. Schmitt N. (San Francisco, CA: Jossey-Bass), 350–400.

[B33] Edwards J. R. Parry M. E. (1993). On the use of polynomial regression equations as an alternative to difference scores in organizational research. *Acad. Manage. J.* 36 1577–1613. 10.5465/256822 256822

[B34] Fleenor J. W. Smither J. W. Atwater L. E. Braddy P. W. Sturm R. E. (2010). Self–other rating agreement in leadership: a review. *Leadersh. Q.* 21 1005–1034. 10.1016/j.leaqua.2010.10.006

[B35] Folger R. Konovsky M. A. (1989). Effects of procedural and distributive justice on reactions to pay raise decisions. *Acad. Manage. J.* 32 115–130. 10.2307/256422

[B36] Gerhart B. Fang M. (2014). Pay for (individual) performance: issues, claims, evidence and the role of sorting effects. *Hum. Resour. Manage. Rev.* 24 41–52. 10.1016/j.hrmr.2013.08.010

[B37] Gibson C. B. Cooper C. D. Conger J. A. (2009). Do you see what we see? The complex effects of perceptual distance between leaders and teams. *J. Appl. Psychol.* 94 62–76. 10.1037/a0013073 19186896

[B38] Graso M. Camps J. Strah N. Brebels L. (2019). Organizational justice enactment: an agent-focused review and path forward. *J. Vocat. Behav.* 116:103296 10.1016/j.jvb.2019.03.007

[B39] Greenberg J. (1993). “The social side of fairness: interpersonal and informational classes of organizational justice,” in *Justice in the Workplace: Approaching Fairness in Human Resource Management*, ed. Cropanzano R. (Hillsdale, NJ: Lawrence Erlbaum Associates, Inc), 79–103.

[B40] Greenberg J. (2001). Setting the justice agenda: seven unanswered questions about “what, why, and how”. *J. Vocat. Behav.* 58 210–219. 10.1006/jvbe.2001.1792

[B41] Greenberg J. (2004). Stress fairness to fare no stress: managing workplace stress by promoting organizational justice. *Organ. Dyn.* 33 352–365. 10.1016/j.orgdyn.2004.09.003

[B42] Hall D. T. Hall F. S. (1976). The relationship between goals, performance, success, self-image, and involvement under different organization climates. *J. Vocat. Behav.* 9 267–278. 10.1016/0001-8791(76)90055-5

[B43] Hasson H. Tafvelin S. Von Thiele Schwarz U. (2013). Comparing employees and managers’ perceptions of organizational learning, health, and work performance. *Adv. Dev. Hum. Resour.* 15 163–176. 10.1177/1523422313475996

[B44] Hasson H. Thiele Schwarz U. Nielsen K. Tafvelin S. (2016). Are we all in the same boat? The role of perceptual distance in organizational health interventions. *Stress Health* 32 294–303. 10.1002/smi.2703 27501357

[B45] Hatfield J. D. Huseman R. C. (1982). Perceptual congruence about communication as related to satisfaction: moderating effects of individual characteristics. *Acad. Manage. J.* 25 349–358. 10.2307/255996

[B46] Hellgren J. Sverke M. Isaksson K. (1999). A two-dimensional approach to job insecurity: consequences for employee attitudes and well-being. *Eur. J. Work Organ. Psychol.* 8 179–195. 10.1080/135943299398311

[B47] Heneman R. L. Ledford G. L. Gresham M. T. (2000). “The changing nature of work and its effects on compensation design and delivery,” in *Compensation in Organization: Current Research and Practice*, eds Rynes S. Gerhart B. (San Francisco, CA: Jossey-Bass), 195–240.

[B48] Holtz B. C. Harold C. M. (2009). Fair today, fair tomorrow? A longitudinal investigation of overall justice perceptions. *J. Appl. Psychol.* 94 1185–1199. 10.1037/a0015900 19702364

[B49] Huang J. L. Li A. Xin-An Z. Cropanzano R. Ping S. Yuhui L. (2017). Employee conscientiousness, agreeableness, and supervisor justice rule compliance: a three-study investigation. *J. Appl. Psychol.* 102 1564–1589. 10.1037/apl0000248 28749156

[B50] Karam E. P. Hu J. Davison R. B. Juravich M. Nahrgang J. D. Humphrey S. E. (2019). Illuminating the ‘Face’ of Justice: a meta-analytic examination of leadership and organizational justice. *J. Manage. Studies* 56 134–171. 10.1111/joms.12402

[B51] Kim J. (2016). Impact of performance appraisal justice on the effectiveness of pay-for-performance systems after civil service reform. *Public Pers. Manage.* 45 148–170. 10.1177/0091026016644625

[B52] Kim S. H. Laffranchini G. Wagstaff M. F. Jeung W. (2017). Psychological contract congruence, distributive justice, and commitment. *J. Manag. Psychol.* 32 45–60. 10.1108/jmp-05-2015-0182

[B53] Koopman J. Scott B. A. Matta F. K. Conlon D. E. Dennerlein T. (2019). Ethical leadership as a substitute for justice enactment: an information-processing perspective. *J. Appl. Psychol.* 104 1103–1116. 10.1037/apl0000403 30843704

[B54] Lawler E. E. (2000). *Rewarding Excellence: Pay Strategies for the New Economy.* San Francisco, CA: Joey-Bass.

[B55] Leventhal G. S. (1980). “What should be done with equity theory? New approaches to the study of fairness in social relationships,” in *Social Exchange: Advances in Theory and Research*, eds Gergen K. Greenberg M. Willis R. (New York, NY: Plenum), 27–55. 10.1007/978-1-4613-3087-5_2

[B56] Levy P. E. Williams J. R. (2004). The social context of performance appraisal: a review and framework for the future. *J. Manage.* 30 881–905. 10.1016/j.jm.2004.06.005

[B57] Lind E. A. (2001). Thinking critically about the justice judgments. *J. Vocat. Behav.* 58 220–226. 10.1006/jvbe.2001.1793

[B58] Maslyn J. M. Uhl-Bien M. (2001). Leader–member exchange and its dimensions: Effects of self-effort and other’s effort on relationship quality. *J. Appl. Psychol.* 86 697–708. 10.1037/0021-9010.86.4.697 11519653

[B59] Moshavi D. Brown F. W. Dodd N. G. (2003). Leader self-awareness and its relationship to subordinate attitudes and performance. *Leadersh. Organ. Dev. J.* 24 407–418. 10.1108/01437730310498622

[B60] Muthén L. K. Muthén B. O. (1998). *Mplus User’s Guide.* Los Angeles, CA: Muthén & Muthén.

[B61] Ostroff C. Shin Y. Kinicki A. J. (2005). Multiple perspectives of congruence: relationships between value congruence and employee attitudes. *J. Organ. Behav.* 26 591–623. 10.1002/job.333

[B62] Patient D. L. Skarlicki D. P. (2010). Increasing interpersonal and informational justice when communicating negative news: the role of the manager’s empathic concern and moral development. *J. Manage.* 36 555–578. 10.1177/0149206308328509

[B63] Podsakoff P. M. Mackenzie S. B. Podsakoff N. P. (2012). Sources of method bias in social science research and recommendations on how to control it. *Annu. Rev. Psychol.* 63 539–569. 10.1146/annurev-psych-120710-100452 21838546

[B64] Podsakoff P. M. Mackenzie S. M. Lee J. Podsakoff N. P. (2003). Common method variance in behavioral research: a critical review of the literature and recommended remedies. *J. Appl. Psychol.* 88 879–903. 10.1037/0021-9010.88.5.879 14516251

[B65] Rupp D. E. Cropanzano R. (2002). The mediating effects of social exchange relationships in predicting workplace outcomes from multifoci organizational justice. *Organ. Behav. Hum. Decis. Process.* 89 925–946. 10.1016/s0749-5978(02)00036-5

[B66] Rupp D. E. Shao R. Jones K. S. Liao H. (2014). The utility of a multifoci approach to the study of organizational justice: a meta-analytic investigation into the consideration of normative rules, moral accountability, bandwidth-fidelity, and social exchange. *Organ. Behav. Hum. Decis. Process.* 123 159–185. 10.1016/j.obhdp.2013.10.011

[B67] Rynes S. L. Gerhart B. Minette K. A. (2004). The importance of pay in employee motivation: discrepancies between what people say and what they do. *Hum. Resour. Manage.* 43 381–394. 10.1002/hrm.20031

[B68] Scott B. A. Colquitt J. A. Paddock L. (2009). An actor-focused model of justice rule adherence and violation: the role of managerial motives and discretion. *J. Appl. Psychol.* 94 756–769. 10.1037/a0015712 19450011

[B69] Shanock L. R. Baran B. E. Gentry W. A. Pattison S. C. Heggestad E. D. (2010). Polynomial regression with response surface analysis: a powerful approach for examining moderation and overcoming limitations of difference scores. *J. Bus. Psychol.* 25 543–554. 10.1007/s10869-010-9183-4

[B70] Shapiro D. (2001). The death of justice theory is likely if theorists neglect the “wheels” already invented and the voices of the injustice victims. *J. Vocat. Behav.* 58 235–242. 10.1006/jvbe.2001.1795

[B71] Sin H.-P. Nahrgang J. D. Morgeson F. P. (2009). Understanding why they don’t see eye to eye: an examination of leader–member exchange (LMX) agreement. *J. Appl. Psychol.* 94 1048–1057. 10.1037/a0014827 19594243

[B72] Sjöberg A. Sverke M. (2000). The interactive effect of job involvement and organizational commitment on job turnover revisited: a note on the mediating role of turnover intention. *Scand. J. Psychol.* 3 247–252. 10.1111/1467-9450.00194 11041307

[B73] Spector P. E. (2006). Method variance in organizational research: truth or urban legend? *Organ. Res. Methods* 9 221–232. 10.1177/1094428105284955

[B74] Sweeney P. D. (1990). Distributive justice and pay satisfaction: a field test of an equity theory prediction. *J. Bus. Psychol.* 4 329–341. 10.1007/bf01125243

[B75] Tafvelin S. Von Thiele Schwarz U. Hasson H. (2017). In agreement? Leader-team perceptual distance in organizational learning affects work performance. *J. Bus. Res.* 75 1–7. 10.1016/j.jbusres.2017.01.016

[B76] Tekleab A. G. Bartol K. M. Liu W. (2005). Is it pay levels or pay rises that matter to fairness and turnover? *J. Organ. Behav.* 26 899–921. 10.1002/job.352

[B77] Tekleab A. G. Taylor M. S. (2003). Aren’t there two parties in an employment relationship? Antecedents and consequences of organization-employee agreement on contract obligations and violations. *J. Organ. Behav.* 24 585–608. 10.1002/job.204

[B78] Thibaut J. Walker L. (1975). *Procedural Justice: A Psychological Analysis.* Hillsdale, NJ: Lawrence Erlbaum Associates.

[B79] Turban D. B. Jones A. P. (1988). Supervisor-subordinate similarity: types, effects, and mechanisms. *J. Appl. Psychol.* 73 228–234. 10.1037/0021-9010.73.2.228 3384773

[B80] Van Dijke M. De Cremer D. Bos A. E. R. Schefferlie P. (2009). Procedural and interpersonal fairness moderate the relationship between outcome fairness and acceptance of merit pay. *Eur. J. Work Organ. Psychol.* 18 8–28. 10.1080/13594320701856699

[B81] Wexley K. N. Alexander R. A. Greenawalt J. P. Couch M. A. (1980). Attitudinal congruence and similarity as related to interpersonal evaluations in manager-subordinate dyads. *Acad. Manage. J.* 23 320–330. 10.5465/255434

[B82] Wu X. Sturman M. C. Wang C. (2013). The motivational effects of pay fairness: a longitudinal study in Chinese star-level hotels. *Cornell Hosp. Q.* 54 185–198. 10.1177/1938965512471891

[B83] Yammarino F. J. Atwater L. E. (1993). Understanding self-perception accuracy: Implications for human resource. *Hum. Resour. Manage.* 32 231–247. 10.1002/hrm.3930320204

[B84] Zapata C. P. Olsen J. E. Martins L. L. (2013). Social exchange from the supervisor’s perspective: employee trustworthiness as a predictor of interpersonal and informational justice. *Organ. Behav. Hum. Decis. Process.* 121 1–12. 10.1016/j.obhdp.2012.11.001

